# Deep learning segmentation of peri-sinus structures from structural magnetic resonance imaging: validation and normative ranges across the adult lifespan

**DOI:** 10.1186/s12987-024-00516-w

**Published:** 2024-02-13

**Authors:** Kilian Hett, Colin D. McKnight, Melanie Leguizamon, Jennifer S. Lindsey, Jarrod J. Eisma, Jason Elenberger, Adam J. Stark, Alexander K. Song, Megan Aumann, Ciaran M. Considine, Daniel O. Claassen, Manus J. Donahue

**Affiliations:** 1 Dept. of Neurology, Vanderbilt University Medical Center, Nashville, TN USA; 2 Dept. of Radiology and Radiological Sciences, Vanderbilt University Medical Center, Nashville, TN USA; 3 Dept. of Psychiatry and Behavioral Sciences, Vanderbilt University Medical Center, Nashville, TN USA; 4 Dept. of Electrical and Computer Engineering, Vanderbilt University, Nashville, TN USA

**Keywords:** Glymphatic, Parasagittal dural space, Arachnoid granulation, CSF flow, Machine learning

## Abstract

**Background:**

Peri-sinus structures such as arachnoid granulations (AG) and the parasagittal dural (PSD) space have gained much recent attention as sites of cerebral spinal fluid (CSF) egress and neuroimmune surveillance. Neurofluid circulation dysfunction may manifest as morphological changes in these structures, however, automated quantification of these structures is not possible and rather characterization often requires exogenous contrast agents and manual delineation.

**Methods:**

We propose a deep learning architecture to automatically delineate the peri-sinus space (e.g., PSD and intravenous AG structures) using two cascaded 3D fully convolutional neural networks applied to submillimeter 3D *T*_2_-weighted non-contrasted MRI images, which can be routinely acquired on all major MRI scanner vendors. The method was evaluated through comparison with gold-standard manual tracing from a neuroradiologist (*n* = 80; age range = 11–83 years) and subsequently applied in healthy participants (*n* = 1,872; age range = 5-100 years), using data from the Human Connectome Project, to provide exemplar metrics across the lifespan. Dice-Sørensen and a generalized linear model was used to assess PSD and AG changes across the human lifespan using quadratic restricted splines, incorporating age and sex as covariates.

**Results:**

Findings demonstrate that the PSD and AG volumes can be segmented using *T*_2_-weighted MRI with a Dice-Sørensen coefficient and accuracy of 80.7 and 74.6, respectively. Across the lifespan, we observed that total PSD volume increases with age with a linear interaction of gender and age equal to 0.9 cm^3^ per year (*p* < 0.001). Similar trends were observed in the frontal and parietal, but not occipital, PSD. An increase in AG volume was observed in the third to sixth decades of life, with a linear effect of age equal to 0.64 mm^3^ per year (*p* < 0.001) for total AG volume and 0.54 mm^3^ (*p* < 0.001) for maximum AG volume.

**Conclusions:**

A tool that can be applied to quantify PSD and AG volumes from commonly acquired *T*_2_-weighted MRI scans is reported and exemplar volumetric ranges of these structures are provided, which should provide an exemplar for studies of neurofluid circulation dysfunction. Software and training data are made freely available online (https://github.com/hettk/spesis).

**Supplementary Information:**

The online version contains supplementary material available at 10.1186/s12987-024-00516-w.

## Introduction

Cerebrospinal fluid (CSF) production occurs with passage of blood through the capillaries and ependymal cells within the choroid plexus complexes, resulting in the production of approximately 500 mL of CSF daily at a rate of 20–25 mL CSF per hour [1]. As the human choroid plexus resides predominately in the lateral ventricles, the majority of CSF produced traverses the cerebral aqueduct en route to the 4th ventricle prior to passage into the subarachnoid space [2, 3]. CSF is ultimately resorbed into the bloodstream through multiple hypothesized pathways [4], including along the olfactory route via the cribriform plate [5, 6], the optic route [7], as well as along peri-sinus structures.

Among these peri-sinus structures are the intravenous arachnoid granulations (AG) which are herniations of the arachnoid membrane that penetrate the dural sinuses and lateral lacunae veins [8–10]. AGs, which have a typical spatial extent of several millimeters to over a centimeter along the longest axis, have been hypothesized to play a fundamental role in CSF regulation. Previous studies have reported that AGs hypertrophy with age [11], and suggested potential relevance to venous hypertension and headaches [12]. However, quantification of these structures generally occurs in *post-mortem* histology studies, or in vivo using manual delineation, with the obvious caveats that *post-mortem* analysis may not accurately reflect in vivo morphology owing to complications of specimen preservation and manual tracings are impractical for routine quantification. In addition, there is accumulating evidence that CSF and trans-arachnoid molecular clearance, as well as immune surveillance and activity, can also occur in the regions surrounding the dural sinuses [13], or the parasagittal dural (PSD) space [14, 15]. This space was initially studied in humans following intrathecal injection of exogenous gadolinium contrast [14], however, this approach is contraindicated in most settings, or impractical for routine surveillance, given the invasive nature of lumbar punctures as well as safety concerns of intraparenchymal gadolinium deposition.

Recently, a non-invasive MRI method was proposed to quantify PSD morphology in humans from high spatial resolution 3D T_2_-weighted MRI and deep learning algorithms [16], whereby it was reported that, using this method, PSD hypertrophies with age and is directly related to the total CSF volume and CSF flow through the cerebral aqueduct. In a separate study using this same method, it was also shown that PSD volume correlates directly with beta-amyloid concentration in older adults with cognitive complaints [17].

These findings collectively support the relevance of accurately characterizing anatomical features of CSF egress, including both AG and PSD volume. Furthermore, these observations highlight the potential relevance of both of these structures in a growing number of pathological conditions where neurofluid circulation dysfunction is being implicated. However, rigorous and automated tools for quantifying these structures non-invasively in vivo from commonly acquired neuroimaging data have not been developed. In this work, we present a new analysis approach for the automatic segmentation of peri-sinus space structures, including both the AG and PSD, based on two cascaded 3D fully convolutional neural networks [18], which we show enables delineation of the PSD and AG solely from non-invasive 3D MRI sequences. The method is demonstrated and validated against gold-standard manual tracings on *T*_2_-weighted imaging, which is routinely acquired on clinical imaging. Additionally, we apply this approach to data available across the lifespan from the Human Connectome Project dataset to provide an exemplar for how PSD and AG change with age and sex in more than 1,000 adults. This work extends the literature by demonstrating that non-invasive MRI can be used to quantify these relevant structures, and we provide the software for free academic use.

## Methods

We first developed and evaluated an automated processing pipeline for PSD and intravenous AG segmentation (https://github.com/hettk/spesis) and subsequently applied this pipeline to Human Connectome Project (HCP) data to evaluate PSD and AG evolution with age and sex across the lifespan.

### Demographics

All participants provided informed consent in accordance with the local institutional review board (IRB). The study included two participant cohorts. First, a prospectively recruited, generalizable adult cohort consisting of persons with and without neurodegeneration (Vanderbilt Glymphatic Imaging Project, VGIP), and second, a larger cohort of participants from the HCP to evaluate the performance of the algorithm on a separate, independent dataset, as well as to calculate exemplar values across the lifespan.

The Vanderbilt Glymphatic Imaging Project dataset (VGIP). All participants (age = 18–83 years) were scanned between January 2020 and September 2021 at Vanderbilt University Medical Center. Inclusion criteria: compatible with 3 Tesla MRI. Exclusion criteria: history of cerebrovascular disease, anemia, psychosis, or neurological disorder including but not limited to prior overt stroke, sickle cell anemia, schizophrenia, bipolar disorder, Alzheimer’s disease, Parkinson’s disease, or multiple sclerosis. The presence of non-specific white matter lesions was not an exclusion criterion, as these lesions are prevalent with normal aging, and we sought our cohort to be representative. Clinical history was reviewed by a board-certified Neurologist (DOC; experience = 14 years) and anatomical imaging and angiography by a board-certified neuroradiologist (CDM; experience = 12 years) to ensure criteria were met.

The Human Connectome Project (HCP). Data were collected from three different sources: HCP young-adult (HCP-YA, age = 22–35 years), HCP aging (age = 36–100 years), and development (age = 5–21 years) [19], named HCP-DA hereon (i.e., combination of HCP development and aging). All participants (*n* = 1,715; age range = 5-100 years) were assessed using phone screenings to rule out major health conditions. HCP excludes participants who have been diagnosed and treated for major psychiatric (e.g., schizophrenia and bipolar disorder) or neurological (e.g., stroke, brain tumors, Parkinson’s disease) disorders as well as individuals with severe depression that required treatment for 12 months or longer in the past five years. The telephone interview for cognitive status was also used to exclude participants with impaired cognitive abilities [20].

### Acquisition

VGIP. All scans were acquired at Vanderbilt University Medical Center using the same 3 Tesla MRI acquisition protocol (Philips Medical Systems, Best, The Netherlands) with body coil radiofrequency transmission and phased array 32-channel SENSE reception. A non-contrasted 3D *T*_2_-weighted (sagittal acquisition) volumetric isotropic turbo-spin-echo acquisition (VISTA) sequence was planned along the anterior commissure– posterior commissure line: field-of-view (anterior-posterior x foot-head x right-left) = 250 × 250 × 188.8 mm, repetition time = 2500 ms, echo time = 331 ms, spatial resolution = 0.78 × 0.78 × 0.78 mm. Additionally, a 3D *T*_1_-weighted MPRAGE scan was acquired with repetition time = 8.1 ms, echo time = 3.7 ms, and spatial resolution = 1 × 1 × 1 mm.

HCP. All scans from the HCP were acquired at 3 Tesla.

Scans collected from HCP-YA were acquired using the customized “Connectome” scanner with 100 mT/m gradient strength. The 3D TSE *T*_2_-weighted sequence was acquired with a repetition time = 2500 ms, echo time = 331 ms, and spatial resolution = 0.8 × 0.8 × 0.8 mm. Additionally, 3D *T*_1_-weighted MPRAGE scans were acquired with a repetition time = 8.1 ms echo time = 3.7 ms, and spatial resolution = 0.7 × 0.7 × 0.7 mm.

HCP-DA scans were acquired from four different sites using a Siemens Prisma scanner at all sites with 80 mT/m gradient coil. Image acquisition applied a 3D variable-flip-angle turbo-spin-echo *T*_2_-weighted sequence (SPACE) with repetition time = 3,200 ms, echo time = 564 ms, and spatial resolution = 0.8 × 0.8 × 0.8 mm, and 3D MPRAGE *T*_1_-weighted sequence with repetition time = 2500 ms, echo time = 3.6 ms, flip angle = 8 degrees, and spatial resolution = 0.8 × 0.8 × 0.8 mm [19, 21].

### Manual delineation of peri-sinus structures

The relevant structures of interest, PSD and intravenous AG, were manually segmented by a board-certified radiologist (CDM) and a diagnostic radiology resident (under CDM supervision) on a cumulative 80 scans from both the VGIP (*n* = 53, age = 18–86) and HCP (*n* = 27, age = 11–80) data sets. Both data sets were used, over a wide age range (i.e., 10 to 86 years old), with the intent of increasing generalizability. Manual delineation has been conducted using the itk-SNAP software (version 3.8.0 and 4.0.0). All *T*_2_-weighted scans were manually delineated in the native space after N4 inhomogeneity correction.

**Fig. 1:**
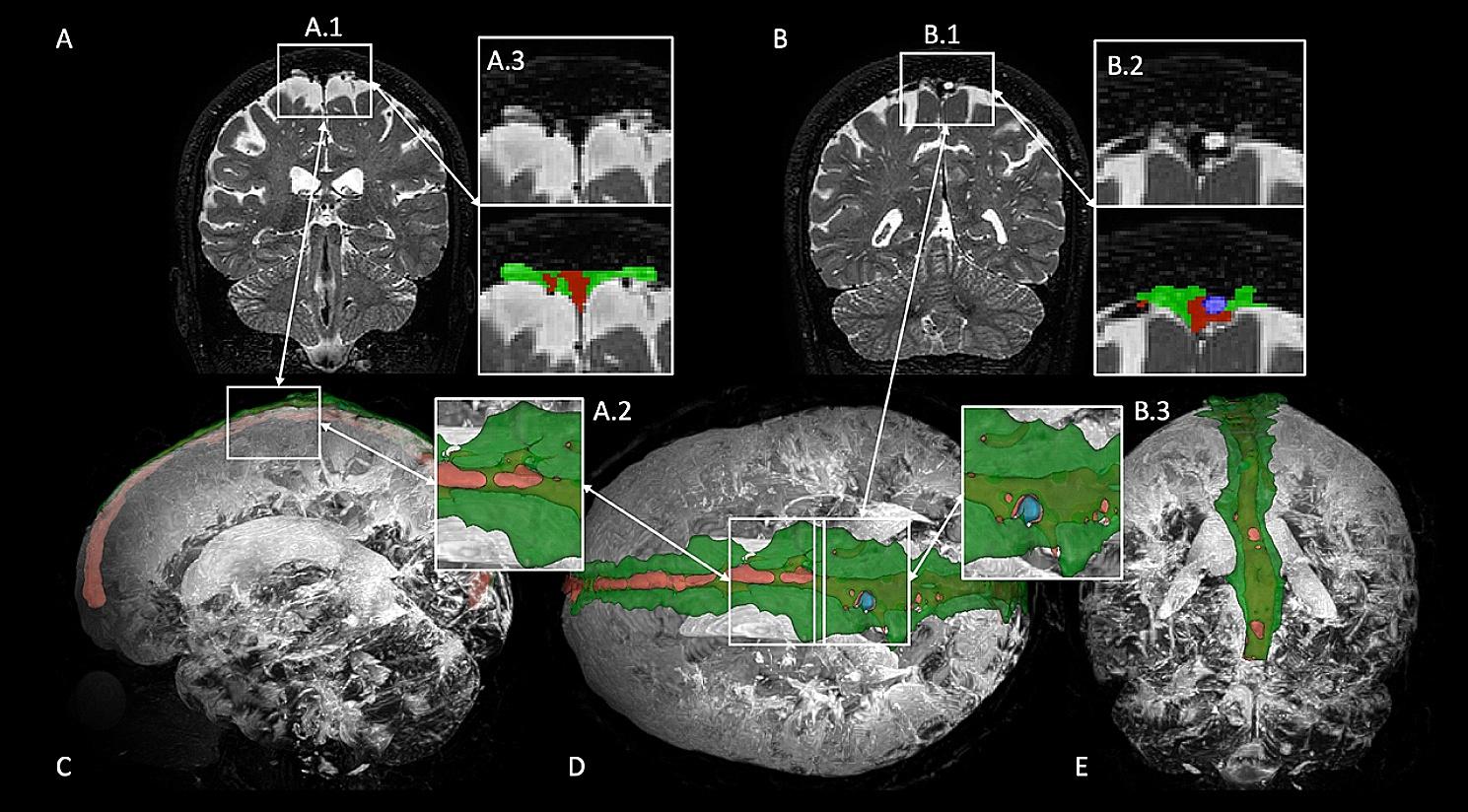
Anatomical depiction of the landmarks used to delineate parasagittal dural (PSD) space (green) and intravenous arachnoid granulations (AG) (blue), venous lumen (red). Sketch illustrating localization and morphology of the peri-sinus structures (**A**). Coronal view displaying medium-sized AG and enlargement of the parasagittal dural space (**B-1,2,3**). Coronal view showing large AG and small enlargement of PSD (**C-1,2,3**). PSD appears as intermediate *T*_2_-weighted signal above a layer of dura mater (i.e., hypointense *T*_2_ signal) and subarachnoid space (i.e., hyperintense *T*_2_ signal)

Both intravenous AG (AG Type I) and parasagittal space were evaluated. The intravenous AGs are CSF-filled structures that extend into the venous sinus. These AGs emerge from the subarachnoid space, extend through the dura mater, and penetrate the superior sagittal sinus lumen and may present with similar *T*_2_-weighted signal intensity as the PSD, however, the structures can be distinguished as they focally protrude into the lumen of the venous sinuses (Fig. 1) whereas the PSD is located along the lateral margins of the superior sagittal sinus. By contrast, the PSD (i.e., merging definition of stromal and diploic AG) demonstrate intermediate *T*_2_-weighted signal intensity relative to the *T*_2_ hyperintense subarachnoid CSF and *T*_2_ hypointense dura mater, superior sagittal sinuses, and calvarium. The medial and superior margins of the PSD are the superior sagittal sinus and the calvarium respectively, which are both relatively *T*_2_ hypointense (Fig. 1). The lateral and inferior margins of the PSD are composed of the opposed dura and arachnoid mater which appear as a linear *T*_2_ hypointense band in the coronal plane. Segmentations were performed up to 30 mm from the midline section following previous postmortem studies reporting extension of lateral lacuna in humans [22].

### Algorithm development

**Fig. 2:**
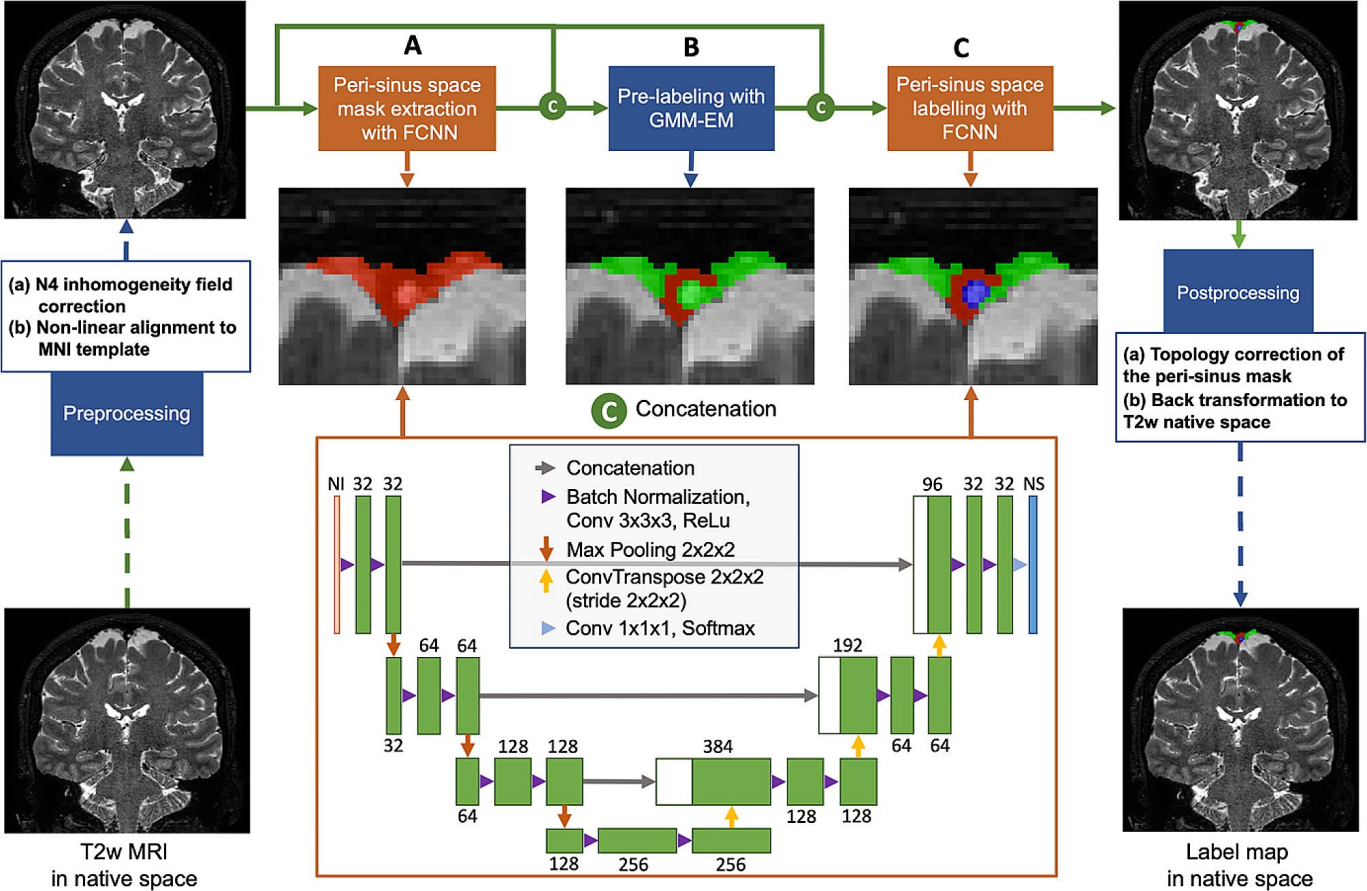
Pipeline of the proposed method; blocks of machine learning appear in orange and non-learning methods appear in blue. Input *T*_2_-weighted MRIs in native space are first preprocessed (N4 bias field inhomogeneity corrected, and registration to a Montreal Neurological Institute (MNI) template using non-rigid transformation), (**A**) The first block of the segmentation method aims to extract the peri-sinus mask (combined background or parasagittal space and arachnoid granulation), (**B**) A is Gaussian mixture model is fit to data using an expectation maximum (EM) algorithm to estimate the maximum a posteriori probability distribution and assign a label for each voxel (i.e., parasagittal dural space or sinus), (**C**) A second U-net is then used to label arachnoid granulation and correct miss-labeled parasagittal dural space voxels. The final label map is transformed back to native space using inverse transform

Following the manual segmentations, we endeavored to design an algorithm to automatically segment PSD and intravenous AG structures. The proposed method was based on a combination of a fully convolutional neural network (F-CNN) and classical model-based method (Fig. 2). First, signal normalization was performed (i.e., spatial, and intra-subject intensity normalization). Second, the first layer of the F-CNN was used to estimate the peri-sinus space mask, followed by pre-labelling of voxels belonging to PSD space and sinus lumen using a gaussian mixture model fitted by the expectation maximization algorithm. This pre-labelling map was then concatenated to the *T*_2_-weighted image and supplied to a second layer of the F-CNN to produce final labelling of the PSD space, intravenous AG, and sinus lumen. Finally, the segmentation was transformed back to *T*_2_-weighted native space within the post-processing step, and an automated report generated (e.g., see *Supplementary Material*). Additional details of this pipeline are provided below.

For pre-processing, intra-subject voxel intensity with N4 inhomogeneity field correction was performed [23]. Next, to reduce anatomical variability all *T*_2_-weighted MRIs were aligned to the MNI template [24] using non-linear registration computed with ANTs [25] (control spacing point = 2 mm). This value provides a good tradeoff between the robustness of the registration (i.e., limitation of eventual registration artifacts) and increase of inter-subject similarity of the peri-sinus space.

The second step of the pipeline consisted of the first F-CNN layer, denoted FCNN_mask_. FCNN_mask_ which delineated peri-sinus space from the subarachnoid space and the remaining brain parenchyma. Of note, peri-sinus space may contain venous blood vessels (superior sinus and afferent veins), dura mater, PSD, and AG. The FCNN_mask_ model was trained using manual binary delineation where all voxels belonging to sinus, PSD, and AG were set to 1, with the remaining voxels assigned to 0.

Once this peri-sinus space mask was estimated using the FCNN_mask_, a gaussian mixture model was fit using an expectation maximization algorithm and an optimal threshold. The maximum *a posteriori* probability was used to assign a label for each voxel belonging to the peri-sinus space mask. Here, PSD space and arachnoid granulations shared the same label, but were separated from the superior sinus.

The readout produced by the pre-labeling step was then concatenated and supplied to the second F-CNN layer noted FCNN_labels_. FCNN_labels_ accepted two inputs and produced a vector composed of four probabilities from the final soft-max layer: background, sinus lumen, PSD, and AG. Final labeled correspond to the maximum probability estimated by FCNN_labels_.

The two F-CNN models employed in the proposed method (i.e., FCNN_mask_ and FCNN_labels_) shared the same architecture, which derives from the 3D U-Net architecture [26] (Fig. 2). U-Net architecture was chosen for its good performance with medical images and limited training data size requirements. Moreover, the peri-sinus structures represent a small component of the entire imagery. It is well-known that over-representation of background can create a bias in learning. In addition, to reduce this potential confound, a patch-based approach was used. Hence, 3D patches of 96 × 64 × 64 voxels were extracted following a peri-sinus probabilistic atlas. The peri-sinus probabilistic atlas was computed using the mean of manual delineation after non-linear registration. Intensity normalization was performed at a patch level, using the z-score method.

The F-CNN was based on a U-Net architecture and comprised an encoding step composed of two blocks of batch normalization, 3D convolutional layer with a kernel set to 3 × 3 × 3 voxels followed by a rectified linear unit (ReLu), in each layer. Between each layer, feature maps were first down sampled using maximum pooling with a kernel of 2 × 2 × 2 voxels. A 3D transposed convolution operator was chosen as the up-sampling method with a kernel set to 2 × 2 × 2 voxels and a stride set to 2 × 2 × 2 voxels. The final segmentation map was obtained using a block composed of a 3D convolution operator with a kernel of 1 × 1 × 1 voxel followed by a soft-max function.

Once the final labeling of the PSD space and AGs were assigned, the final peri-sinus space mask was corrected to ensure its topology (i.e., remove non-contiguous regions) using a closing operation from mathematical morphology set of techniques [27]. Finally, the segmentation mask was transformed back to the native *T*_2_-weighted space using an inverse transformation and nearest-neighbor interpolation.

As peri-sinus space structures can have different trajectories dependent on their locations along the superior sinus, sub-segments of peri-sinus space were drawn in the MNI-ICBM 152 template and aligned to the subject space. Sub-segments were defined according to frontal, parietal, and occipital portions. The parietal region was delineated from the frontal PSD using the central sulcus and extended to the parietal–occipital fissure; finally, the occipital PSD was delineated from the parietal–occipital fissure to the most posterior portion of the PSD.

### Implementation

Random flipping along the sagittal fissure was performed during the training phase to address limited training data size. Noise injection was performed using randomly assigned standard deviation of a random variable draw from a normal distribution. A contrast limited adaptive histogram equalization (CLAHE) augmentation was used to increase the generalization capability of the model.

Both networks, FCNN_mask_ and FCNN_labels_, were trained using the ADAM optimizer with a learning rate set to 10^− 4^. First, the FCNN_mask_ was trained using a manual delineation (MD) set as (MD > 0) and cross entropy as loss functions,

Eq. 1 $$ {L}_{CE}=-{\sum }_{i=1}{t}_{i}\text{log}\left({p}_{i}\right)$$

where $$ {t}_{i} $$represents the ground truth label and $$ {p}_{i}$$ represents the estimated probability computed using the first network. Second, different from the training of FCNN_mask_, a generalized dice loss functions ($$ {L}_{GDSC}$$) was used to train the second network, FCNN_labels_ [28],

Eq. 2 $$ {L}_{GDSC}= 1-2\frac{{\sum }_{l}{w}_{l}{\sum }_{n}{r}_{ln}{p}_{ln} }{{\sum }_{l}{w}_{l}{\sum }_{n}{r}_{ln}{p}_{ln}}$$

where, $$ {r}_{ln} $$and $$ {p}_{ln}$$ represent the ground truth and estimated labels, respectively. $$ {w}_{l}$$ represents the weight variable computed as $$ {w}_{l}=1/\left({\sum }_{n}{r}_{ln}\right)$$. Generalized dice loss has been used to address the unbalanced nature of structures size.

### Evaluation

A cross-validation scheme was used to evaluate the proposed method. The 80 manually labeled scans were separated into three distinct groups: training, validation, and testing. Using a pseudo-random assignment, the training set was composed of 60 scans, the validation set was composed of 10, and the testing set was composed of 10. This process was iterated eight times to obtain unbiased segmentation for all scans.

As proposed in [29], we evaluated the segmentation accuracy for each structure of interest (i.e., PSD and AG) using a set of commonly incorporated metrics. First, to evaluate the global overlap between estimated segmentation and ground truth, we computed the Dice-Sørensen coefficient (DSC). Furthermore, we evaluated segmentation using recall and precision.

Finally, given the sparse characteristic of the AG component compared to PSD space which is a single structure running along the superior sinus, and to evaluate the feasibility to assess arachnoid granulation metrics, we proposed to calculate the confidence of estimating total number, total volume, average volume, and maximum volume, compared to manual delineation using Pearson’s correlation coefficient and root mean squared difference (RMS).

### Statistics and hypothesis testing

The segmentation performance was evaluated in two ways. First, the method was applied across different cohorts to evaluate generalizability. Second, the domain generalization was considered by comparing the segmentation results of the method trained on the VGIP and the HCP dataset (i.e., two independent cohorts acquired at different sites on different scanner vendors). For both analyses, segmentation metrics were separately evaluated for the PSD space and AG. Performance metrics between methods were evaluated using unpaired t-tests.

Next, we applied the segmentation method to quantify PSD and AG volumes across the lifespan in the larger HCP dataset. PSD volume and AG volume were assessed as percentage of intracranial volume (ICV). ICV was estimated using the *T*_1_-weighted scans as input to AssemblyNet [30], a recent ensemblist deep-learning software for the segmentation of brain structures. ICV characterizes the total brain volume (e.g., gray and white matter parenchyma) in addition to the CSF. Linear mixed effect models were used to assess changes in PSD and AG volume across the adult human lifespan. Peri-sinus structure metrics (PSD volumes, AG volumes, and AG count) were used as separate dependent variables and age and sex as covariates as described; the interactions between age and sex were modeled using quadratic restricted splines with the source dataset used as a random variable (i.e., VGlP, HCP-YA, and HCP-DA). Goodness-of-fit was assessed using adjusted R^2^ and Akaike information criterion (AIC) scores.

## Results

### Evaluation of segmentation accuracy

Table 1 summarizes the automatic segmentation from the proposed method compared to that of manual tracing from a radiologist. The proposed method provided high accuracy of the PSD structure with DSC = 80.7%, recall = 84.7%, precision = 84.9%, and RMS = 0.66 cm^3^. Total PSD volumes demonstrated a strong correlation with manual segmentation with a Pearson’s correlation score of 0.96 (*p* < 0.001). Subdivision of the PSD volume yielded higher accuracy in the frontal aspect with an average DSC equal to 83.9% and a Pearson’s correlation of 0.96 (*p* < 0.001). The method demonstrated lower, albeit still acceptable, accuracy in the parietal and occipital regions with DSC = 80.6% and 79.4%, respectively, and a correlation with manual tracing = 0.95 and 0.87 (*p* < 0.001).

**Table 1:** Evaluation of arachnoid granulation labelling over different interest metrics and comparison to gold standard manual segmentation. Results provided are means with standard deviations in parentheses. The method accuracies are evaluated using Dice-Sørensen coefficients (DSC), Recall, and Precision. Root mean square difference (RMS) and Pearson correlation (R) were computed using estimated volume and manual tracing

	DSC (std)	Recall (std)	Precision (std)	RMS (std)	R (*p*-value)
Total	80.7 (11.4)	84.7 (11.4)	84.9 (0.0)	0.66 (0.44)	0.96 (< 0.001)
Frontal	83.9 (15.3)	86.7 (15.3)	84.9 (0.0)	0.33 (0.25)	0.96 (< 0.001)
Parietal	80.6 (15.7)	91.5 (15.7)	86.3 (0.0)	0.22 (0.18)	0.95 (< 0.001)
Occipital	79.4 (23.4)	89.2 (23.4)	94.6 (11.9)	0.15 (0.14)	0.87 (< 0.001)

The second evaluation consisted of assessing the feasibility of automatically detecting AGs by combining a non-contrasted MRI sequence and machine learning (Table 2). The proposed method provided delineation of the AG structure with an average DSC = 74.6%, recall = 67.3%, precision = 83.6%, and RMS = 21.2mm^3^. Total AG volumes demonstrated a strong correlation with manual segmentation with a Pearson’s correlation score of 0.97 (*p* < 0.001). Subdivision of the AG volume showed automatic delineation in the frontal aspect with an average DSC = 71.7% and a Pearson’s correlation = 0.95 (*p* < 0.001). The automatic delineation in the parietal region results in a DSC = 73.9% and a fair correlation with manual tracing of DSC = 0.96 (*p* < 0.001). No AG was observed in the occipital lobe, therefore, evaluation metrics in this region are not reported.

**Table 2:** Evaluation of arachnoid granulation labelling over different interest metrics and comparison to gold standard manual segmentation. Results provided are averages (standard deviation). The method accuracies are evaluated using Dice-Sørensen coefficients (DSC), Recall, and Precision. Root mean square difference (RMS), expressed in mm^3^, and Pearson correlation (R) were computed using estimated volume and manual tracing

	DSC (std)	Recall (std)	Precision (std)	RMS (std)	R (*p*-value)
Total	74.6 (29.6)	67.3 (29.6)	83.6 (30.2)	21.2 (37.5)	0.97 (< 0.001)
Frontal	71.7 (32.1)	71.2 (32.1)	84.8 (35.9)	11.23 (27.56)	0.95 (< 0.001)
Parietal	73.9 (34.9)	67.7 (20.6)	82.4 (41.9)	9.48 (17.78)	0.96 (< 0.001)
Occipital	94.5 (15.2)	94.0 (17.5)	96.2 (12.5)	3.32 (17.39)	0.97 (< 0.001)

Results of PSD and AG segmentation are illustrated in Fig. 4, which display case-examples of automatic segmentation provided by the proposed method compared to the manually delineated segmentation maps along the spectrum of the distribution performance (i.e., 25, 50, 75, and 95 percentiles).

Next, we evaluated the relationship between AG volume estimated by manual tracing and our method (see Table 3). The four measures of interest were total AG volume, number of AGs detected (i.e., count), average AG volume, maximum AG volume, and minimum AG volume (i.e., volume of the smallest detected AG). Findings suggest that the method enables the estimation of total AG volume and AG count with a strong correlation with manual tracing, with a Pearson’s correlation coefficient of 0.97 and 0.90 (*p* < 0.001) with an RMS equal to 21.27 mm^3^ and 1.17 mm^3^, with average AG volume and maximum AG yielding a high correlation with a Pearson’s coefficient of 0.80 (*p* < 0.001) and RMS equal to 3.83 mm^3^. The smallest estimated AG volume (i.e., minimum volume) obtained a correlation coefficient with manual tracing of 0.82 (*p* < 0.001) and a RMS equal to 1.09 mm^3^. The largest AG volume within each individual (i.e., maximum volume) obtained a correlation coefficient with manual tracing of 0.80 (*p* < 0.001) and a RMS equal to 12.83 mm^3^.

**Table 3:** Evaluation of arachnoid granulation labelling over different metrics of interest and comparison to gold standard manual segmentation. Results are expressed using Pearson’s correlation coefficient, uncorrected *p*-values are reported in parentheses, and root mean square (RMS) difference (standard deviation in parenthesis). The RMS is expressed in mm^3^ for all volumetric measures (i.e., total, average, maximum, and minimum), and by number of occurrences for count

	Total Volume	Count	Average volume	Maximum volume	Minimum volume
R (*p*-value)	0.97 (< 0.001)	0.90 (< 0.001)	0.80 (< 0.001)	0.80 (< 0.001)	0.82 (< 0.001)
RMS (std)	21.27 (37.57)	1.17 (1.46)	3.83 (6.11)	12.83 (26.32)	1.09 (0.86)

**Table 4:** Evaluation of segmentation accuracy of PSD and AG in different dataset (i.e., VGlP, HCP-DA, and HCP-YA). The method accuracies are evaluated using Dice-Sørensen coefficients (DSC), Recall, and Precision. Root mean squared (RMS) differences are expressed in cm^3^ for the PSD volume and mm^3^ for the AG volume

		DSC (std)	Recall (std)	Precision (std)	RMS (std)	R (*p*-value)
PSD	VGlP	80.3 (4.3)	82.4 (5.6)	78.4 (4.4)	0.61 (0.42)	0.95 (< 0.001)
	HCP-YA	79.5 (3.4)	82.5 (3.2)	77.0 (5.4)	0.58 (0.44)	0.97 (< 0.001)
	HCP-DA	77.3 (3.8)	82.7 (4.5)	76.2 (2.6)	1.11 (0.31)	0.88 (< 0.001)
AG	VGlP	76.6 (13.4)	75.1 (16.4)	79.5 (12.3)	26.60 (43.96)	0.97 (< 0.001)
	HCP-YA	75.5 (12.1)	75.5 (13.2)	81.1 (7.7)	10.57 (12.74)	0.98 (< 0.001)
	HCP-DA	69.9 (15.6)	69.9 (16.3)	80.4 (15.7)	9.50 (10.68)	0.98 (< 0.001)

Finally, Table 4 summarizes the evaluation of the segmentation accuracy as performed for each data source in order to assess whether the origin of the data may influence the quantified metrics. The segmentation of PSD from individuals enrolled in VGlP and HCP-YA is similar in terms of performance with a DSC of 80.3 and 79.5, respectively. However, a lower, albeit still similar, segmentation accuracy was observed in scans from HCP-DA with an average DSC of 77.3. The same trends were observed for the AG. Scans collected from VGlP and HCP-YA demonstrate similar accuracy with a DSC of 76.6 and 75.5, respectively, and a slightly lower accuracy in the HCP-DA dataset with a DSC of 69.9.

**Fig. 3:**
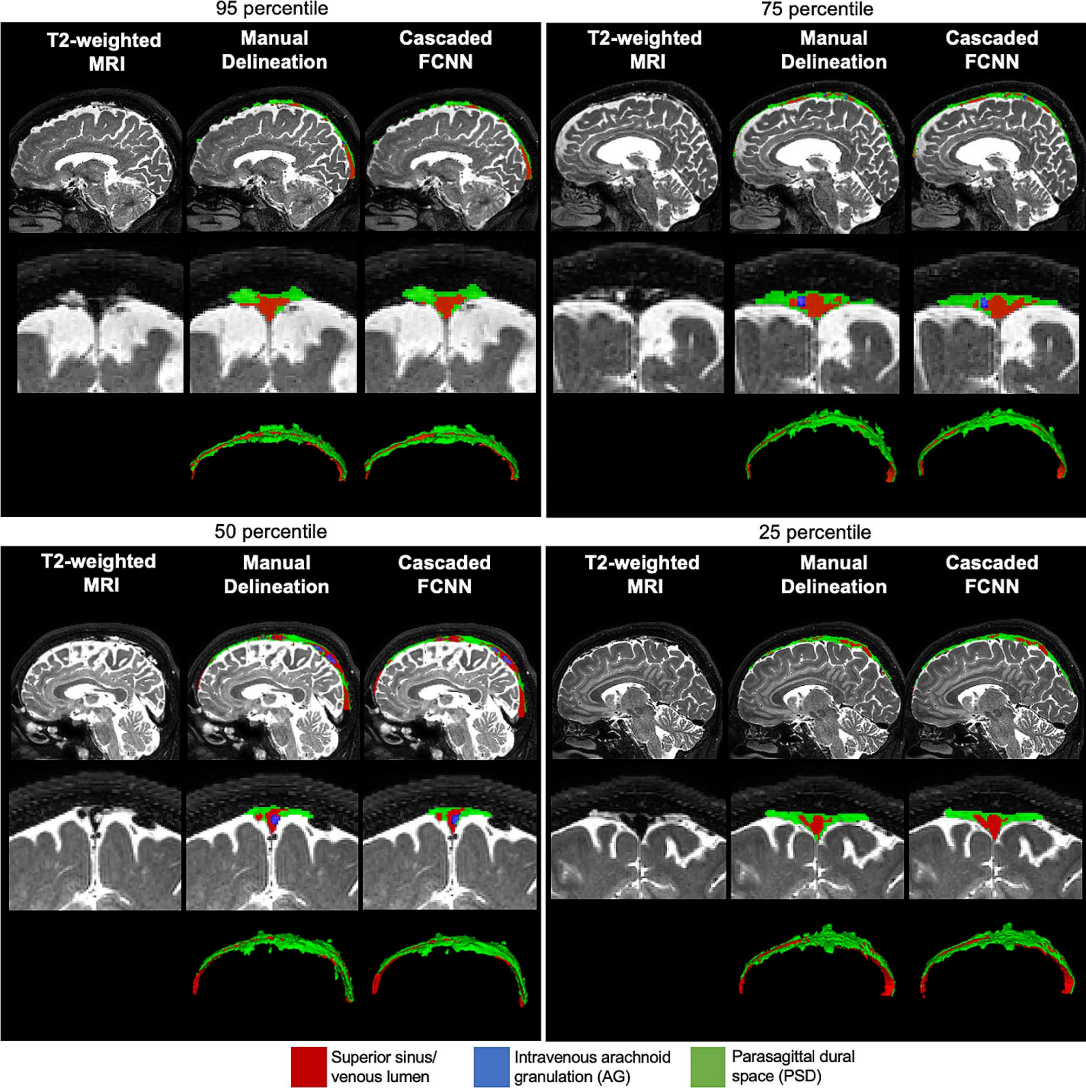
Comparison of segmentation of parasagittal dural space (PSD) and arachnoid granulation (AG) between manual segmentation from a board-certified neuroradiologist, and the proposed method. In each panel, an exemplar case of segmentation for the 95th, 75th, 50th, and 25th percentiles of the distribution of segmentation accuracy as measured by the Dice coefficient is shown

### Evaluation of the volumetrics in the human lifespan

**Fig. 4:**
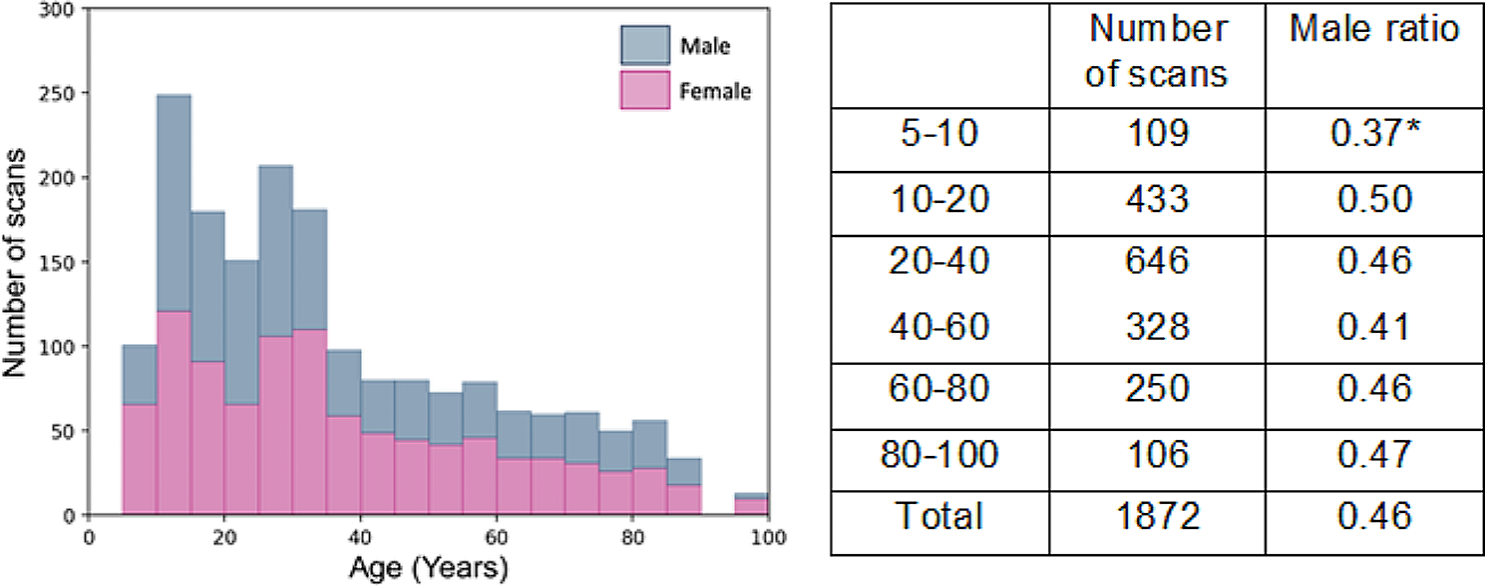
Demographic schematic of the dataset from the open-access Human Connectome Project used to model parasagittal dural space and arachnoid granulation volumes across the human lifespan. The pink color represents the proportion of females, the blue color represents the proportion of male participants, and the bar height represents the total number of scans for each age group. *p-value < 0.05 using a chi-square test which assesses difference of proportion between female and male in each age segment

**Fig. 5:**
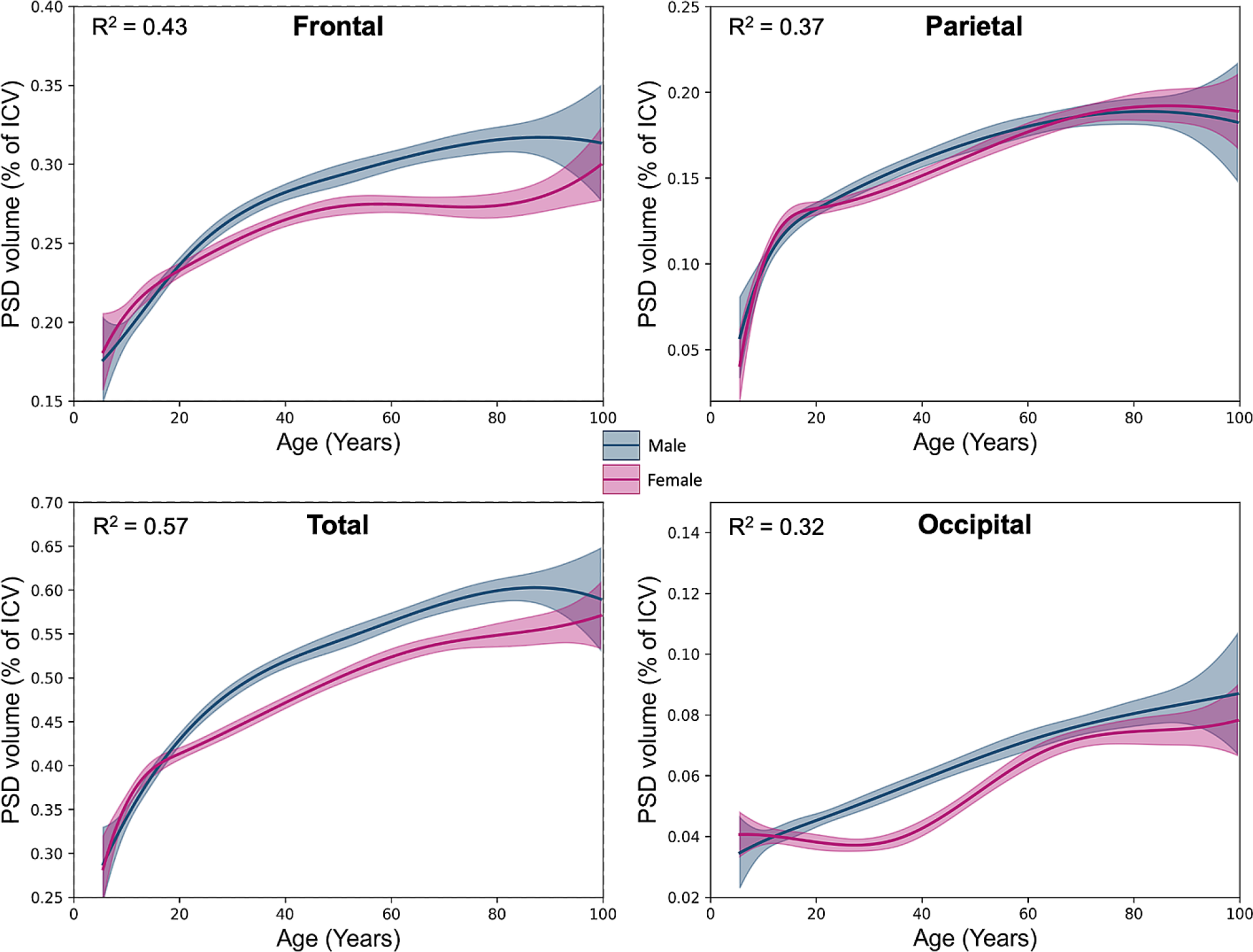
Modelling of the parasagittal dural (PSD) space volumes in each region of interest (i.e., total, frontal, parietal, and occipital) using restricted quadratic spline models. Blue and purple curves represent average PSD volume in male and female, respectively. Gray curves represent average of PSD volume for both genders

Next, we evaluated peri-sinus structures volume in the human lifespan using a large cohorts of subjects aged 5-100 years. In total, 1,872 scans composed of 861 male and 1011 female participants, were used for following analyses (see age distribution and sex proportion in Fig. 4). Figure 5 shows average PSD volume over the human lifespan. Quadratic models indicate an increase of PSD volume as total volume and sub-regions. Total PSD volume increases with age are more pronounced in males compared to females with a linear interaction of gender and age equal to 0.9 cm^3^ per year (*p* < 0.001). Total PSD volume reached a plateau near 70 years of age with an average PSD volume reaching 8 cm^3^ in male participants compared to 6.5 cm^3^ in female participants. Similar trends occur in the frontal and parietal regions.

**Fig. 6:**
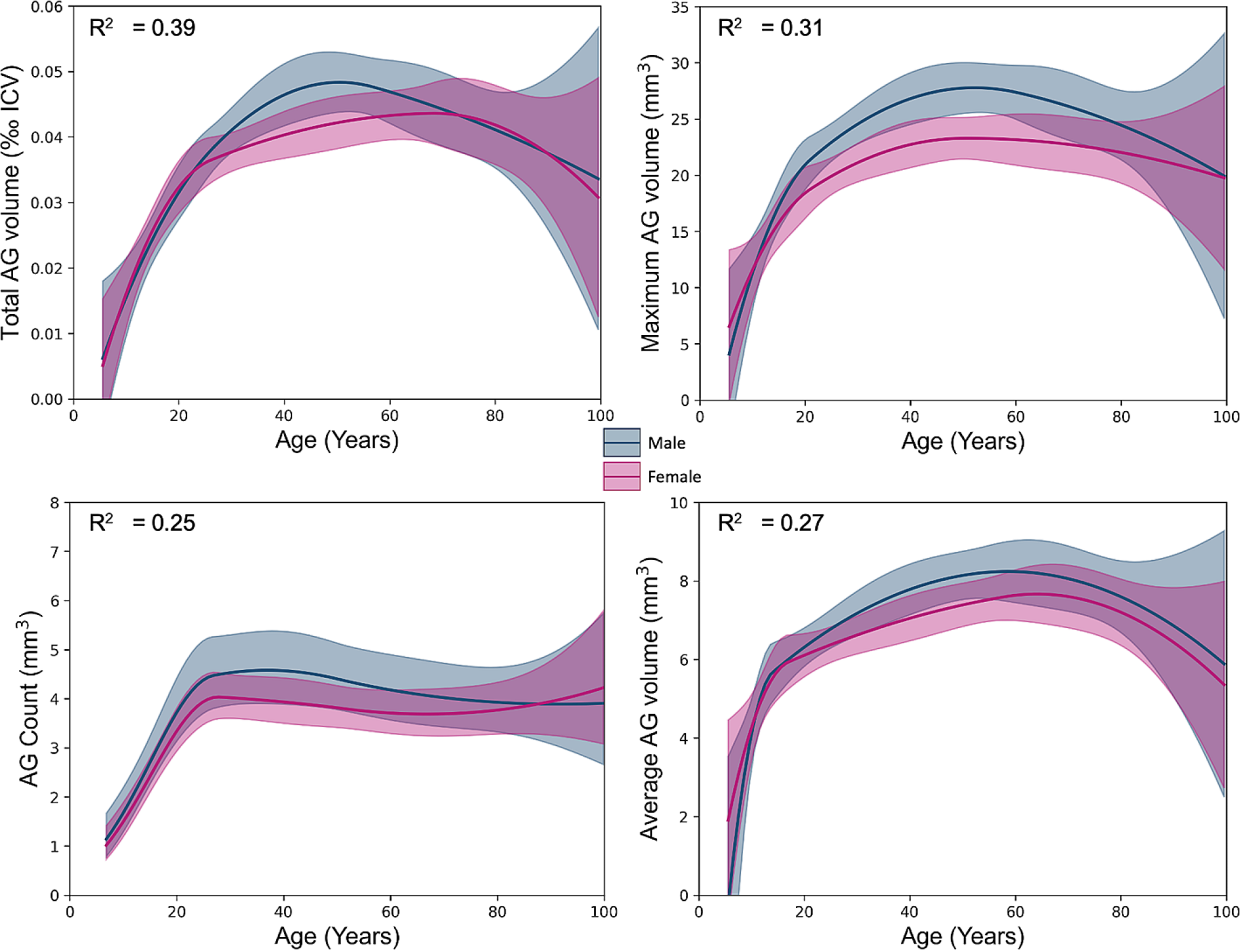
Modelling of the total arachnoid granulation (AG) volume in each metrics of interest (i.e., total, maximum, average volume, and number) using restricted quadratic spline models. Blue and purple curves represent average AG measure in male and female, respectively. Gray curves represent average of AG measures for both genders

Figure 6 shows evolution of proposed AG metrics across the human lifespan. An increase of AG volume was observed in the third to sixth decades of life, with a linear effect of age equal to 0.64 mm^3^ per year (*p* < 0.001) for total AG volume, and 0.54 (*p* < 0.001) for maximum AG volume, and one new AG detected every four years during the first two decades of life (*p* < 0.001).

## Discussion

We propose a new process to quantify PSD and intravenous AG volumes, which have recently been implicated in CSF egress or neuroimmune surveillance. The proposed method enables the automatic extraction of the PSD and AGs from non-invasive 3D *T*_2_-weighted MRI, which should render it easily implementable in standard clinical and research protocols. Furthermore, we applied our new method in a larger dataset of 1,872 scans to assess volumetric changes in PSD and AG across the typically developing human lifespan.

### Methodologic evaluation

The in vivo visualization of human PSD space was first shown using intrathecal injection of gadolinium-based contrast agents [14]. However, this technique requires injection of contrast directly into the intrathecal space, which is deemed unethical and contraindicated for most research studies in the United States. Following this seminal work, a separate study proposed to use intravenous (IV) injection of gadobutrol to quantify changes in the PSD space in melanoma patients [31]. In this work, Park et al. used post-contrasted *T*_1_-weighted black blood MRI combined with a thresholding method to discriminate the PSD space. As exogenous contrast agents are becoming increasingly restricted in research studies [32, 33], non-invasive methods are more widely relevant and provide a complementary approach. In prior work, we investigated the use of non-contrasted MRI sequence to quantify PSD volume [16]. Here, we combined a deep-learning technique to extract peri-sinus space and proposed to delineate PSD space from the superior sagittal sinus lumen. It is noteworthy that in this first approach, AGs were not differentiated from PSD volumes. The current method detailed here enables the added delineation of the AGs and addresses the two major limitations of methods using gadolinium as a contrast agent to highlight PSD space. The use of *T*_2_-weighted MRI (a) increases the generalizability as this sequence can be readily added to non-contrasted head MRI protocols and (b) bypasses any safety concerns related to downstream effects of gadolinium contrast administration. Given safety concerns with intrathecal gadolinium injections, it was not possible to compare our results directly with intrathecal contrast studies, and rather, we used manual delineation of the parasagittal dural space, by a board-certified neuroradiologist, for gold-standard validation.

Similarly, we evaluated for the first time the performance of intravenous AG segmentation and compared metrics estimated from these segmentation masks to gold-standard manual segmentation performed by a board-certified neuroradiologist. This experiment showed a moderate segmentation accuracy for the three investigated populations of participants in terms of overlap between segmentation masks automatically estimated by our method and manual delineation. Of the multiple AG metrics, the detection of the largest AG (i.e., maximum volume) and total volume of AG were the most reliable measurements when comparing to manual neuroradiologist segmentation. AG limitations derive from two factors that must be addressed in future work to increase the reliability of arachnoid granulation segmentation. First, a better definition of structure features must be established. Although this aspect is not emphasized in this work, a stricter definition of arachnoid granulation is fundamental to enable deep learning to learn specific patterns of such structure. Second, AG delineation is associated with large intra- and inter-subject spatial variability. Therefore, modeling this structure requires a robust algorithm that will accommodate variable anatomical patterns (e.g., shape, contrast, size, and localization). Additional increase of the size for the training set will also improve model performance. This is the subject of ongoing investigation.

It should also be noted that this work focused on identifying the PSD space and intravenous AG from a single non-invasive MRI sequence, specifically a 3D *T*_2_-weighted sequence with a spatial resolution of 0.8–0.9 mm isotropic. Incorporation of additional MRI acquisitions, such as FLAIR and magnet resonance venography may help to further disambiguate the PSD from surrounding venous structures and AG which may increase the performance of the automated segmentation further.

The results of both methods compared to manual delineation are illustrated in Fig. 3 for different levels of segmentation performance. This illustrates the robustness of segmentation quality provided by the method. 95, 75, and 50 percentile of the performance indicates that the proposed method provides a similar estimation of the PSD space. The additional FCNN layer enables us a better estimation of the PSD space in regions with lower *T*_2_-weighted signal contrast and accurately delineates AGs. In these two examples, the method was able to detect AGs as small as 2 mm^3^. This represents, in the spatial resolution of the native *T*_2_-weighted scan, a volume of 3 to 4 voxels. Such results motivate future studies investigating changes of intravenous AG and this as well is the topic of ongoing work.

### Integration in the current literature

Initially characterized as a principal site for CSF egress, the role of the perisinusoidal drainage and AGs evolved following studies that utilized intrathecal injection of gadolinium into the CSF [14]. Somewhat surprisingly, the contrast agent attained its maximum concentration in the plasma before reaching peak levels in peri-sinusoidal structures [15]. This implicates alternative efflux pathways as primary loci for the clearance of cerebrospinal fluid and metabolic waste. While the findings from these studies build upon the current comprehension of CSF egress, the details of these pathways are still not entirely understood.

It was recognized that gadolinium-based contrast demonstrates trans-arachnoid passage from the subarachnoid space at the vertex to the structures in the peri-sinus space (e.g., PSD and AG). However, the distinctive egress patterns of contrast may be influenced by the molecular properties of the particular gadolinium-based contrast agent. Additionally, these studies involved the injection of a tracer into the spinal subarachnoid space, which is far removed from the superior sinus regions where AGs are prominent. Consequently, the tracer may have followed divergent egress pathways more anatomically proximate to the lumbar injection site prior to reaching the peri-sinus structures such as AGs.

### PSD and AG across the human lifespan and future directions

One useful aspect of the proposed methodology is that it can be applied to commonly acquired non-invasive brain MRIs, as are acquired in large consortium datasets such as the HCP. This allows for new insights into how these structures evolve across the lifespan which would not be possible from focal, single-site studies with smaller samples. As previous linear modeling of age and sex effect suggested [15, 16, 31], our experiments indicate an increase of PSD volume related to age. Improvement of our model enables us to detect a faster increase in males compared to females in the first half of life. Of note, male participants have an average similar PSD volume in early life compared to female participants. The findings suggest that differentiation of PSD volume between males and females may occur during childhood and adolescence, which could be related to distinct developmental processes. This highlights the need to evaluate CSF dynamics in youth to understand how structures related to CSF production and egress evolve during brain development [34]. Moreover, analysis of regional growth of PSD volume indicates that, on average, enlargement of the PSD begins in the frontal region and progresses to the parietal and occipital regions later in life.

In terms of AGs, the same trends have been shown in human lifespan analysis, with an increase of AG volume and AG number more pronounced in the first three decades of life, with in average one new AG detected each decade of life. These findings follow a previous study investigating changes in AG volume and number [11]. The decrease of average AG metrics along with increased confidence interval after 60 years of life is noteworthy. Several factors may contribute and warrant future attention. For instance, the sample size after age 60 years is restricted, even more so after age 80 years, which may contribute to larger confidence intervals. One potential contributory process to this decrease in sample size is a survival bias, whereby increasing rates of exclusionary neurological conditions reduces the available asymptomatic control sample in later decades. Relatedly, the apparent parabolic increase and then decrease of AG metrics centered around the late-50s / early-60s may represent a modeling artifact. Specifically, there may be pathological AG hypertrophy emerging near age 50 years in a subset of the HCP presumed asymptomatic control group, reflecting a new early-biomarker or prodromal stage of neurodegenerative disease, yet these participants may not meet HCP diagnostic criteria for exclusion. This would not be surprising considering the non-comprehensive neurological and neuropsychological screening necessitated by their large-scale methodological approach. After approximately an additional 5–10 years, this type of participant manifests sufficiently to be excluded. When this pattern is combined with a proposed healthy aging pattern involving a plateau of AG metrics at midlife, it would result in a smoothed inverse parabola, as observed here. Replicating the analyses of this study in a much larger sample of adults aged 50 years and older who have undergone comprehensive neurological and neuropsychological evaluation, with longitudinal follow-up, would bolster confidence in the normal developmental trajectory of AG and PSD morphology in later life. Cross-sectional application of the same analyses in well-established neurodegenerative disease populations would lend further confidence to delineating potentially normal age-related changes across different decades of adulthood in these metrics from those related to neuropathologies with progressively later age-of-onset (i.e., Huntington’s disease, Parkinson’s disease, and Alzheimer’s disease). Longitudinal application in those at risk of developing these conditions could then evaluate AG and PSD metrics utility as a novel early structural biomarker of neurodegeneration. Such studies are needed to understand the potential usage of arachnoid granulation quantification for monitoring and predicting the development of neurological conditions.

Future studies such as those investigating the relationship of white matter hyper-intensities (WMh) and brain cisternal volumes with the peri-sinus structures could provide insight on the PSD and AG functions. Such lesions, despite often being considered non clinically meaningful when diffuse and burden low, may reflect inflammation resulting from the damage of the axonal myelin sheath [35] or chronic mild ischemia which could, in principle, influence perivascular flow profiles. Investigating, the relationship between PSD and AG in context of these lesions could provide further support of the neuroinflammation interface model as recently proposed [15] and the algorithms developed here should be easily applicable to such future studies. Additionally, CSF is localized to both ventricular and subarachnoid compartments and the composition of fluid in these compartments, and distribution between these compartments, may vary in the setting of neurofluid circulation dysfunction. These variations could relate to regional differences in the egress pathway, which also can be investigated using the methods developed here to better characterize egress pathways.

### Limitations

The study findings should also be considered considering several limitations. Initially, it is essential to highlight that the existing version of the deep-learning model underwent training with a dataset comprising participants aged 11 to 83 years old. Subsequently, the methodology was applied to a broader age range to extrapolate structural volumes across a slightly wider demographic spectrum. An ad hoc analysis (presented in Supplemental Materials) indicates that, despite the high accuracy of estimations in older age groups (i.e., 80 to 100 years old), the current iteration of our method tends to overestimate PSD volume in very young cohorts (i.e., 5–10 years old). Although this overestimation does not alter the observed volume trajectory reported in our analysis, this underscores the imperative to enhance the current model to rectify this limitation by performing additional model training on datasets from young children with gold standard manual tracings; this will be the topic of future work. Second, we develop an automated segmentation method for peri-sinus structures using high-resolution *T*_2_-weighted MRI while most recent studies investigating this structure reported higher contrast for PSD structure using fluid-attenuated inversion recovery (FLAIR). However, FLAIR sequences are designed to suppress the longitudinal magnetization from CSF, which reduces contrast in CSF-filled structures such as AGs. Therefore, visualization of AG is suboptimal when using FLAIR scans, while T_2_-weighted MRI provides a good tradeoff between the high contrast on signals from the PSD and intravenous AG. Third, the lower average volume observed in late life in AG structures could be reflective of the inner limitation of cross-sectional study. Future longitudinal investigations are required to understand volumetric changes of peri-sinus structures at an individual level. Despite this limitation, analyses presented from our study provides normative range in the human lifespan. Finally, we validated our method using manual tracing from a neuroradiologist, which is generally considered the gold-standard for validation purposes. It would have been additionally useful to compare findings with segmentation from intrathecal gadolinium injections, however, this protocol is simply deemed unethical at our, and most, hospitals. As we provide our algorithm free for public use, investigators at other centers may be in a better position to make such a comparison.

## Conclusions

We propose a new method for the automatic segmentation of peri-sinus space including delineation of the parasagittal dural space and intravenous arachnoid granulations. This method provides a new tool to study a site of CSF egress, which may have relevance to fluid and waste clearance related to the recently proposed glymphatic circuit and is intended to enable researchers to study such structures with only the use of non-contrasted *T*_2_-weigthed MRI. Moreover, findings further enable investigation of morphological changes occurring in the parasagittal dural spaces and CSF egress pathways in the setting of multiple neurologic disorders. The application of this method in a large cohort of participants across the lifespan provides normative ranges for comparison in pathological conditions.

### Electronic supplementary material

Below is the link to the electronic supplementary material.

Supplementary Material 1

## Data Availability

The data that support the findings of this study are available from the corresponding author, MJD, upon reasonable request. The segmentation software is freely available for public use at: https://github.com/hettk/spesis.

## Abbreviations

AG: Arachnoid granulation
ANTs: Advanced Normalization Tools
CSF: Cerebrospinal fluid
FCNN: Fully convolutional neural network
FLAIR: Fluid attenuated inversion recovery
FSL: FMRIB Software Library
HCP: Human Connectome Project
HCP-YA: Human Connectome Project Young-Adult
HCP-DA: Human Connectome Project Development-Aging
ICBM-MNI: International Consortium for Brain Mapping-Montreal Neurological Institute
ICV: Intracranial volume
IRB: Institutional Review Board
MCI: Mild cognitive impairment
MPRAGE: Magnetization-prepared-rapid-gradient-echo
MRI: Magnetic resonance imaging/images
PET: Positron emission tomography
PSD: Parasagittal dural
RMS: Root-mean-square
SENSE: Sensitivity encoding
TE: Echo time
TR: Repetition time
VGIP: Vanderbilt Glymphatic Imaging Project
VISTA: Volumetric isotropic turbo-spin-echo acquisition
