# Supplementary material for: Deep learning segmentation of peri-sinus structures from structural magnetic resonance imaging: validation and normative ranges across the adult lifespan
Source: Fluids Barriers CNS. 2024 Feb 13;21:15. doi: 10.1186/s12987-024-00516-w (PMC10865560; doi:10.1186/s12987-024-00516-w)
Supplement: Supplementary file 1 — Supplementary Material 1 [file 12987_2024_516_MOESM1_ESM.docx]

**Deep learning segmentation of peri-sinus structures from structural magnetic resonance imaging: validation and normative ranges across the adult lifespan**

Kilian Hett^1^, Colin D. McKnight^2^, Melanie Leguizamon^1^, Jennifer S. Lindsey^2^, Jarrod J. Eisma^1^, Jason Elenberger^1^, Adam J. Stark^1^, Alexander K. Song^1^, Megan Aumann^1^, Ciaran M. Considine^3^, Daniel O. Claassen^1^, and Manus J. Donahue^1,3,4*^

^1^Vanderbilt University Medical Center, Dept. of Neurology, Nashville, TN, USA

^2^Vanderbilt University Medical Center, Dept. of Radiology, Nashville, TN, USA

^3^Vanderbilt University Medical Center, Dept. of Psychiatry, Nashville, TN, USA

^4^Vanderbilt University, Dept. of Electrical and Computer Engineering, Nashville, TN, USA

Prepared for submission as an original research article in *Fluids and Barriers of the CNS*

**Supplementary materials**

**Model accuracy analyses**

**Table 1.** Measure of mask overlap in the pediatric cohort (age=5-10 years). The method accuracies are evaluated using Dice-Sørensen coefficients (DSC), Recall, and Precision. Fidelity of the volume estimation was estimated using Pearson’s correlation coefficient; uncorrected p-values are reported in parentheses along with root mean square (RMS) difference (standard deviation in parenthesis). The RMS is expressed in cm^3^ for the PSD and mm^3^ for AG. Experiments indicate good overlap between ground truth and estimated label mask for the PSD. However, the current model led to a systematic overestimation of the volume on an order of 2 cm^3^ above the measured volume. It is also noteworthy that AGs are extremely rare, or beyond the detection threshold, in pediatric brains and therefore, accuracy measured as mask overlap should be interpreted with caution. Measures of accuracy over the volume estimation indicates high fidelity of automatic estimation with estimation performed by manual delineation.

|  | **DSC (std)** | **Recall (std)** | **Precision (std)** | **RMS (std)** | **R (p-value)** |
| --- | --- | --- | --- | --- | --- |
| **PSD** | 77.4 (12.0) | 90.5 (8.3) | 68.3 (14.15) | 1.82 (1.12) | 0.49 (0.40) |
| **AG** | 40.0 (48.9) | 100.0 (0.0) | 40.0 (48.9) | 2.00 (4.00) | 0.90 (0.04) |

**Table 2.** Measure of mask overlap in the geriatric cohort (age=85-100 years). The method accuracies are evaluated using Dice-Sørensen coefficients (DSC), Recall, and Precision. Fidelity of the automatic estimation of volume was estimated using Pearson’s correlation coefficient; uncorrected p-values are reported in parentheses along with root mean square (RMS) difference (standard deviation in parenthesis). The RMS is expressed in cm^3^ for the PSD and mm^3^ for AG. As a result of larger PSD and AG structures in geriatric brains, which happen to be more similar to data contained in the training set, analysis of segmentation performance demonstrates high overlap between manually and automatically delineated masks. Similar with mask overlap, the proposed method estimates PSD and AG volumes with high fidelity.

|  | **DSC (std)** | **Recall (std)** | **Precision (std)** | **RMS (std)** | **R (p-value)** |
| --- | --- | --- | --- | --- | --- |
| **PSD** | 92.1 (6.2) | 93.9 (5.4) | 90.5 (7.0) | 0.34 (0.28) | 0.96 (0.01) |
| **AG** | 89.1 (12.0) | 84.6 (16.3) | 95.3 (5.9) | 22.73 (30.44) | 0.95 (0.01) |

**Lifespan analyses**

Parasagittal dural space


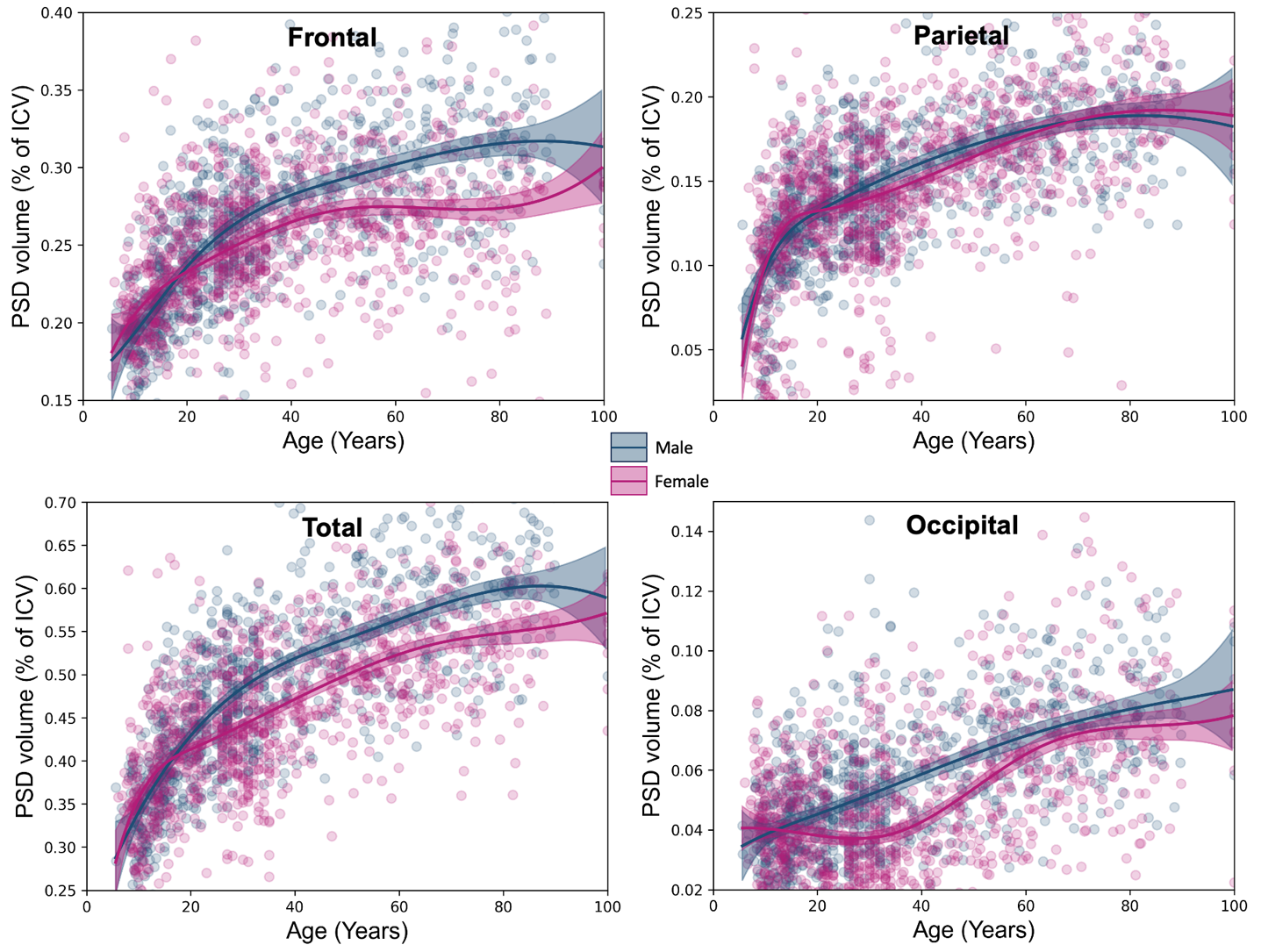


**Figure 1.** Modelling of the parasagittal dural (PSD) space volumes in each region of interest (i.e., total, frontal, parietal, and occipital) using restricted quadratic spline models. Blue and purple curves represent average PSD volume in male and female, respectively. Gray curves represent average of PSD volume for both genders.

**Table 3.** Summary of linear mixed effect model fitting for the total PSD volume. Knots = [20,52] in male and [20,67] in female.

|  | **Coefficient** | **Std. Error** | **t** | **p** | **[0.025** | **0.975]** |
| --- | --- | --- | --- | --- | --- | --- |
| **Male** | 0.2876 | 0.021 | 13.420 | 0.000 | 0.246 | 0.330 |
| **Female** | 0.2824 | 0.019 | 14.913 | 0.000 | 0.245 | 0.319 |
| **cs(Age)[0]:Male** | 0.0626 | 0.029 | 2.179 | 0.029 | 0.006 | 0.119 |
| **cs(Age)[1]:Male** | 0.2082 | 0.020 | 10.299 | 0.000 | 0.169 | 0.248 |
| **cs(Age)[2]:Male** | 0.2636 | 0.032 | 8.294 | 0.000 | 0.201 | 0.326 |
| **cs(Age)[3]:Male** | 0.3383 | 0.028 | 12.191 | 0.000 | 0.284 | 0.393 |
| **cs(Age)[4]:Male** | 0.3009 | 0.039 | 7.779 | 0.000 | 0.225 | 0.377 |
| **cs(Age)[0]:Female** | 0.1072 | 0.025 | 4.214 | 0.000 | 0.057 | 0.157 |
| **cs(Age)[1]: Female** | 0.1544 | 0.018 | 8.661 | 0.000 | 0.119 | 0.189 |
| **cs(Age)[2]: Female** | 0.2725 | 0.029 | 9.525 | 0.000 | 0.216 | 0.329 |
| **cs(Age)[3]: Female** | 0.2665 | 0.023 | 11.674 | 0.000 | 0.222 | 0.311 |
| **cs(Age)[4]: Female** | 0.2893 | 0.028 | 10.370 | 0.000 | 0.235 | 0.344 |
| **Group** | 0.000 | 0.005 |  | | | |

**Table 4.** Summary of linear mixed effect model fitting for the parietal PSD volume. Knots = [20,50] in both male and female.

|  | **Coefficient** | **Std. Error** | **t** | **p** | **[0.025** | **0.975]** |
| --- | --- | --- | --- | --- | --- | --- |
| **Male** | 0.1759 | 0.014 | 12.928 | 0.000 | 0.149 | 0.203 |
| **Female** | 0.1811 | 0.012 | 14.641 | 0.000 | 0.157 | 0.205 |
| **cs(Age)[0]:Male** | 0.0174 | 0.018 | 0.950 | 0.342 | 0.019 | -0.053 |
| **cs(Age)[1]:Male** | 0.0933 | 0.013 | 7.274 | 0.000 | 0.068 | 0.118 |
| **cs(Age)[2]:Male** | 0.1231 | 0.020 | 6.103 | 0.000 | 0.084 | 0.163 |
| **cs(Age)[3]:Male** | 0.1486 | 0.018 | 8.355 | 0.000 | 0.114 | 0.184 |
| **cs(Age)[4]:Male** | 0.1373 | 0.024 | 5.657 | 0.000 | 0.090 | 0.185 |
| **cs(Age)[0]:Female** | 0.0329 | 0.017 | 1.913 | 0.056 | -0.001 | 0.067 |
| **cs(Age)[1]: Female** | 0.0627 | 0.012 | 5.426 | 0.000 | 0.040 | 0.085 |
| **cs(Age)[2]: Female** | 0.1151 | 0.018 | 6.394 | 0.000 | 0.080 | 0.150 |
| **cs(Age)[3]: Female** | 0.0744 | 0.015 | 4.879 | 0.000 | 0.045 | 0.104 |
| **cs(Age)[4]: Female** | 0.1197 | 0.018 | 6.766 | 0.000 | 0.085 | 0.154 |
| **Group** | 0.000 | 0.005 |  | | | |

**Table 5.** Summary of linear mixed effect model fitting for the parietal PSD volume. Knots = [20,57] in male and [20, 69] in female.

|  | **Coefficient** | **Std. Error** | **t** | **p** | **[0.025** | **0.975]** |
| --- | --- | --- | --- | --- | --- | --- |
| **Male** | 0.0569 | 0.012 | 4.722 | 0.000 | 0.033 | 0.081 |
| **Female** | 0.0408 | 0.011 | 3.800 | 0.000 | 0.020 | 0.062 |
| **cs(Age)[0]:Male** | 0.0577 | 0.016 | 3.612 | 0.000 | 0.026 | 0.089 |
| **cs(Age)[1]:Male** | 0.0897 | 0.011 | 7.868 | 0.000 | 0.067 | 0.112 |
| **cs(Age)[2]:Male** | 0.1287 | 0.018 | 7.184 | 0.000 | 0.094 | 0.164 |
| **cs(Age)[3]:Male** | 0.1365 | 0.015 | 8.963 | 0.000 | 0.107 | 0.166 |
| **cs(Age)[4]:Male** | 0.1252 | 0.022 | 5.589 | 0.000 | 0.081 | 0.169 |
| **cs(Age)[0]:Female** | 0.0888 | 0.014 | 6.178 | 0.000 | 0.061 | 0.117 |
| **cs(Age)[1]: Female** | 0.0900 | 0.010 | 8.883 | 0.000 | 0.070 | 0.110 |
| **cs(Age)[2]: Female** | 0.1527 | 0.016 | 9.351 | 0.000 | 0.121 | 0.185 |
| **cs(Age)[3]: Female** | 0.1529 | 0.013 | 11.817 | 0.000 | 0.128 | 0.178 |
| **cs(Age)[4]: Female** | 0.1479 | 0.016 | 9.341 | 0.000 | 0.117 | 0.179 |
| **Group** | 0.000 | 0.001 |  | | | |

**Table 6.** Summary of linear mixed effect model fitting for the occipital PSD volume. Knots = [20,57] in male and [20, 69] in female.

|  | **Coefficient** | **Std. Error** | **t** | **p** | **[0.025** | **0.975]** |
| --- | --- | --- | --- | --- | --- | --- |
| **Male** | 0.0346 | 0.006 | 5.849 | 0.000 | 0.023 | 0.046 |
| **Female** | 0.0406 | 0.004 | 10.944 | 0.000 | 0.033 | 0.048 |
| **cs(Age)[0]:Male** | 0.0052 | 0.008 | 0.642 | 0.521 | -0.011 | 0.021 |
| **cs(Age)[1]:Male** | 0.0167 | 0.006 | 2.964 | 0.003 | 0.006 | 0.028 |
| **cs(Age)[2]:Male** | 0.0415 | 0.009 | 4.477 | 0.000 | 0.023 | 0.060 |
| **cs(Age)[3]:Male** | 0.0485 | 0.008 | 6.458 | 0.000 | 0.034 | 0.063 |
| **cs(Age)[4]:Male** | 0.0524 | 0.012 | 4.257 | 0.000 | 0.028 | 0.077 |
| **cs(Age)[0]:Female** | 0.0006 | 0.007 | 0.086 | 0.932 | -0.013 | 0.014 |
| **cs(Age)[1]: Female** | -0.0140 | 0.004 | -3.678 | 0.000 | -0.021 | -0.007 |
| **cs(Age)[1]: Female** | 0.0416 | 0.007 | 6.002 | 0.000 | 0.028 | 0.055 |
| **cs(Age)[3]: Female** | 0.0317 | 0.006 | 5.223 | 0.000 | 0.020 | 0.044 |
| **cs(Age)[4]: Female** | 0.0378 | 0.007 | 5.180 | 0.000 | 0.023 | 0.052 |
| **Group** | 0.000 | 0.000 |  | | | |

Arachnoid granulation


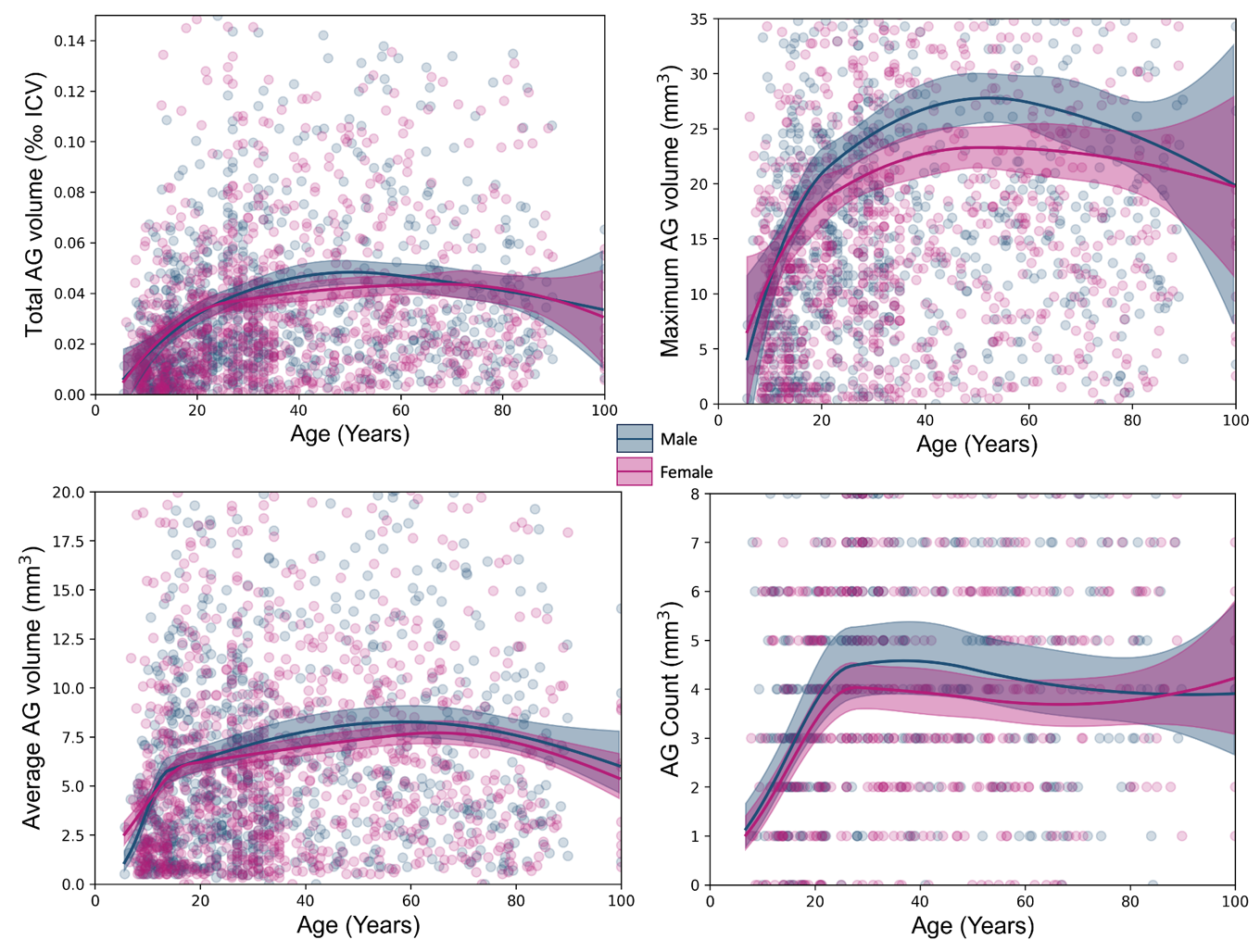


**Figure 2.** Modelling of the total arachnoid granulation (AG) volume in each metrics of interest (i.e., total, maximum, average volume, and number) using restricted quadratic spline models. Blue and purple curves represent average AG measure in male and female, respectively. Gray curves represent average of AG measures for both genders.

**Table 7.** Summary of linear mixed effect model fitting for the total AG volume. Knots = [26,57] in male and [26, 68] in female.

|  | **Coefficient** | **Std. Error** | **t** | **p** | **[0.025** | **0.975]** |
| --- | --- | --- | --- | --- | --- | --- |
| **Male** | 0.0062 | 0.006 | 1.029 | 0.303 | -0.006 | 0.018 |
| **Female** | 0.0051 | 0.005 | 0.979 | 0.328 | -0.005 | 0.015 |
| **cs(Age)[0]:Male** | 0.0230 | 0.009 | 2.557 | 0.011 | 0.005 | 0.041 |
| **cs(Age)[1]:Male** | 0.0449 | 0.006 | 7.415 | 0.000 | 0.033 | 0.057 |
| **cs(Age)[2]:Male** | 0.0365 | 0.010 | 3.754 | 0.000 | 0.017 | 0.056 |
| **cs(Age)[3]:Male** | 0.0272 | 0.013 | 2.110 | 0.035 | 0.002 | 0.053 |
| **cs(Age)[0]:Female** | 0.0280 | 0.008 | 3.612 | 0.000 | 0.013 | 0.043 |
| **cs(Age)[1]: Female** | 0.0383 | 0.005 | 7.136 | 0.000 | 0.028 | 0.049 |
| **cs(Age)[2]: Female** | 0.0387 | 0.008 | 4.768 | 0.000 | 0.023 | 0.055 |
| **cs(Age)[3]: Female** | 0.0253 | 0.011 | 2.375 | 0.018 | 0.004 | 0.046 |
| **Group** | 0.000 | 0.001 |  | | | |

**Table 8.** Summary of linear mixed effect model fitting for the maximum AG volume. Knots = [21,60] in male and [21, 50] in female.

|  | **Coefficient** | **Std. Error** | **t** | **p** | **[0.025** | **0.975]** |
| --- | --- | --- | --- | --- | --- | --- |
| **Male** | -3.4672 | 6.543 | -0.530 | 0.596 | -16.291 | 9.357 |
| **Female** | -0.3898 | 5.852 | -0.067 | 0.947 | -11.860 | 11.081 |
| **ICV** | 0.0051 | 0.004 | 1.408 | 0.159 | -0.002 | 0.012 |
| **cs(Age)[0]:Male** | 14.1318 | 5.051 | 2.798 | 0.005 | 4.232 | 24.032 |
| **cs(Age)[1]:Male** | 24.9120 | 3.643 | 6.838 | 0.000 | 17.772 | 32.052 |
| **cs(Age)[2]:Male** | 21.2190 | 5.354 | 3.963 | 0.000 | 10.725 | 31.713 |
| **cs(Age)[3]:Male** | 15.3383 | 7.295 | 2.103 | 0.035 | 1.041 | 29.636 |
| **cs(Age)[0]:Female** | 11.3497 | 4.550 | 2.495 | 0.013 | 2.433 | 20.267 |
| **cs(Age)[1]: Female** | 16.9120 | 2.923 | 5.785 | 0.000 | 11.183 | 22.641 |
| **cs(Age)[2]: Female** | 16.6452 | 4.520 | 3.683 | 0.000 | 7.786 | 25.504 |
| **cs(Age)[3]: Female** | 13.3300 | 5.105 | 2.611 | 0.009 | 3.324 | 23.336 |
| **Group** | 6.494 | 1.356 |  | | | |

**Table 9.** Summary of linear mixed effect model fitting for the mean AG volume. Knots = [14,54] in male and [18, 58] in female.

|  | **Coefficient** | **Std. Error** | **t** | **p** | **[0.025** | **0.975]** |
| --- | --- | --- | --- | --- | --- | --- |
| **Male** | 0.0814 | 0.256 | 0.319 | 0.750 | -0.419 | 0.582 |
| **Female** | 0.9261 | 0.158 | 5.846 | 0.000 | 0.616 | 1.237 |
| **ICV** | -1.572e-07 | 8.03e-05 | -0.002 | 0.998 | -0.000 | 0.000 |
| **cs(Age)[0]:Male** | 1.6104 | 0.250 | 6.449 | 0.000 | 1.121 | 2.100 |
| **cs(Age)[1]:Male** | 1.9931 | 0.224 | 8.898 | 0.000 | 1.554 | 2.432 |
| **cs(Age)[2]:Male** | 2.0650 | 0.247 | 8.359 | 0.000 | 1.581 | 2.549 |
| **cs(Age)[3]:Male** | 1.7076 | 0.255 | 6.703 | 0.000 | 1.208 | 2.207 |
| **cs(Age)[0]:Female** | 0.8442 | 0.141 | 5.998 | 0.000 | 0.568 | 1.120 |
| **cs(Age)[1]: Female** | 1.0238 | 0.110 | 9.292 | 0.000 | 0.808 | 1.240 |
| **cs(Age)[2]: Female** | 1.1853 | 0.137 | 8.657 | 0.000 | 0.917 | 1.454 |
| **cs(Age)[3]: Female** | 0.7494 | 0.152 | 4.944 | 0.000 | 0.452 | 1.046 |
| **Group** | 6.494 | 1.356 |  | | | |

**Table 10.** Summary of linear mixed effect model fitting for the AG number. Knots = [20,57] in male and [20, 69] in female.

|  | **Coefficient** | **Std. Error** | **t** | **p** | **[0.025** | **0.975]** |
| --- | --- | --- | --- | --- | --- | --- |
| **Male** | -0.3613 | 0.273 | -1.324 | 0.186 | -0.896 | 0.174 |
| **Female** | -0.4782 | 0.249 | -1.922 | 0.055 | -0.966 | 0.010 |
| **ICV** | 0.0005 | 0.000 | 3.708 | 0.000 | 0.000 | 0.001 |
| **cs(Age)[0]:Male** | 1.3219 | 0.242 | 5.471 | 0.000 | 0.848 | 1.795 |
| **cs(Age)[1]:Male** | 1.4206 | 0.167 | 8.516 | 0.000 | 1.094 | 1.748 |
| **cs(Age)[2]:Male** | 1.2024 | 0.233 | 5.151 | 0.000 | 0.745 | 1.660 |
| **cs(Age)[3]:Male** | 1.2323 | 0.243 | 5.067 | 0.000 | 0.756 | 1.709 |
| **cs(Age)[0]:Female** | 1.3974 | 0.217 | 6.428 | 0.000 | 0.971 | 1.823 |
| **cs(Age)[1]: Female** | 1.3622 | 0.152 | 8.945 | 0.000 | 1.064 | 1.661 |
| **cs(Age)[2]: Female** | 1.2301 | 0.208 | 5.918 | 0.000 | 0.823 | 1.638 |
| **cs(Age)[3]: Female** | 1.4282 | 0.213 | 6.714 | 0.000 | 1.011 | 1.845 |
| **Group** | 4.236 | 1.123 |  | | | |
